# Clinical characteristics and outcomes of Langerhans cell histiocytosis at a single institution in Thailand: a 20-year retrospective study

**DOI:** 10.2478/abm-2021-0022

**Published:** 2021-08-20

**Authors:** Ponrachet Kitticharoenjit, Nucharin Supakul, Piya Rujkijyanont, Chanchai Traivaree, Apichat Photia, Chalinee Monsereenusorn

**Affiliations:** Department of Pediatrics, Phramongkutklao Hospital, Bangkok 10400, Thailand; Department of Radiology and Imaging Science, Riley Hospital for Children, Indiana University School of Medicine, Indianapolis, IN 46202, United States of America; Division of Hematology–Oncology, Department of Pediatrics, Phramongkutklao Hospital and Phramongkutklao College of Medicine, Bangkok 10400, Thailand

**Keywords:** fluorodeoxyglucose F18, hyperbilirubinemia, conjugated, hematopoietic, hypoalbuminemia, organs at risk, positron emission tomography computed tomography

## Abstract

**Background:**

Langerhans cell histiocytosis (LCH) is a rare disease characterized by the various systems involved and clinical manifestations with a wide range of symptoms.

**Objectives:**

To describe clinical characteristics, imaging, treatment, and outcomes of pediatric LCH at Phramongkutklao Hospital, Bangkok, Thailand.

**Methods:**

We conducted a 20-year retrospective review of the medical records of patients diagnosed with LCH from birth to 21 years old from January 1, 1997, to December 31, 2016.

**Results:**

In all, 14 patients with median age of 2.5 years were studied. Six (43%) patients had single-system (SS) LCH. Five patients (63%) with multisystem (MS) LCH (n = 8. 57%) had risk-organ involvement (RO+). All patients had plain X-ray imaging of their skull with 11 (79%) showing abnormal findings. Tc-99m bone imaging and fluorodeoxyglucose F18 (FDG) positron emission tomography (PET)-computed tomography (CT) demonstrated abnormal findings in 8 (89%) and 4 (29%) patients, respectively. The 5-year event-free survival (EFS) for patients with RO+ MS-LCH was less than that for those without risk-organ involvement (RO−) MS-LCH and SS-LCH (20% vs. 100%, *P* = 0.005). Hematological dysfunction, hypoalbuminemia, and conjugated hyperbilirubinemia may be worse prognostic factors for RO+ MS-LCH.

**Conclusion:**

FDG-PET-CT might have a greater accuracy to detect LCH disease than conventional plain X-ray and Tc-99m bone imaging. RO+ MS-LCH has been encountered with relapse and poor outcomes. Hematopoietic involvement, hypoalbuminemia, and conjugated hyperbilirubinemia may be worse prognostic factors for RO+ MS-LCH.

Langerhans cell histiocytosis (LCH) is a disease originating from abnormal proliferation of CD1a^+^/CD207^+^ myeloid dendritic cells and characterized by activation of the mitogen-activated protein kinase (MAPK)/extracellular signal-regulated kinase (ERK) signaling pathway [1, 2]. For almost 60%–70% of patients, the somatic activating mutation in the proto-oncogene for the serine/threonine-protein kinase B-Raf or *BRAF*^V600E^ [3, 4], is associated with disseminated disease leading to inferior outcomes [5].

The incidence of LCH among patients aged <15 years was 4–10 per million people in Western populations [6, 7], which is consistent with the <6 per million people annually in Thailand [8]. The childhood cancer registries covered approximately 22% of the Thai population. Fifty percent of those registered were in Bangkok [9]. Phramongkutklao Hospital, a tertiary referral center for childhood cancer in Bangkok, Thailand, constitutes 5%–10% of childhood cancer care in Bangkok. Therefore, there are 1–2 patients newly diagnosed with LCH annually at our institute.

Etiology and risk factors for developing LCH are unclear [10]. Population-based cancer studies have observed a higher incidence in Hispanic, but lower in Black populations, densely-populated countries, and among those with low educational status [6].

LCH has a wide range of symptoms from self-resolution to disseminated disease. Various clinical manifestations have a systemic involvement [11] and all organs can be affected in any age group. Bone is the most affected system and presents in approximately 80% of patients [12]. However, head-to-toe organ involvement can be influenced by the disease [13]. Therefore, prompt and comprehensive evaluation is needed to provide accurate diagnosis and avoid delayed treatment.

Histopathology with positive cluster of differentiation antigen (CD) 1a or CD 207 (Langerin) staining lesional cells, or both, are required to diagnose LCH. Currently, there is no specific laboratory marker or imaging to diagnose LCH. However, radiological diagnosis is mandatory to obtain diagnosis before providing a tissue diagnosis. A radiograph of the skull or Tc-99m bone imaging, or both, are evaluated in patients suspected with LCH, in particular, those presenting with bone lesions. Brain magnetic resonance imaging (MRI) was evaluated in patients presented with diabetes insipidus (DI) [11]. Currently, fluorodeoxyglucose F18 (FDG) positron emission tomography (PET)-computed tomography (CT) is established as an investigation of choice for LCH patients [14].

The standard treatment of LCH is adapted by group-system involvement of the disease [15]. Patients with involvement of a single system (SS) can often be treated with surgery or local therapy, and even spontaneous regression has occurred under close monitoring [16]. For multisystem (MS) involvement, a challenge exists to achieve disease remission, especially for risk-organ involvement (RO+), for which an intensified chemotherapy (CMT) regimen would be warranted and still carries a dismal prognosis [17]. However, groups without organ of risk involvement (RO−) have responded well to prolonged low dose CMT and yet can experience disease reactivation [18]. Similarly, it often ensues with SS multifocal bone (MFB) [19].

To date, few studies conducted in Asian countries have reported the clinical characteristics and outcomes of LCH. Most studies were from China [20, 21]. However, LCH treatment regimens mostly based on studies from Japan [22, 23], have been well documented and have been tolerable for patients in Thailand. Due to the rarity of LCH the clinical characteristics, management, treatment regimens, and outcomes of disease have not been explored fully in pediatric populations. Therefore, the present study of LCH explicitly in Thai children is warranted.

The present study aimed to identify clinical characteristics, investigations, treatment regimens, and outcomes of pediatric LCH in Phramongkutklao Hospital. The results of this study should provide better comprehension of the LCH situation and further facilitate treatment plans, which might apply to our institution and be applied throughout Thailand, and possibly by ASEAN neighbors.

## Methods

### Study population

We conducted a 20-year retrospective review of the medical records of 14 pediatric patients diagnosed with LCH from birth to 21 years old from January 1, 1997 to December 31, 2016. This study was approved by the Ethics Committee and Institutional Review Board of Phramongkutklao Hospital and Phramongkutklao College of Medicine, Bangkok, Thailand, (approval No. IRBRTA 111/2562) and was conducted in accordance with the principles of the Declaration of Helsinki, the guidance of the Council for International Organizations of Medical Sciences (CIOMS), and International Council on Harmonisation of Technical Requirements for Registration of Pharmaceuticals for Human Use (ICH) and good clinical practice (GCP). This observational study has been registered with the Thai Clinical Trials Registry (TCTR https://www.thaiclinicaltrials.org/), with number TCTR20200413001. We included data from patients in whom the diagnosis of LCH was confirmed by histopathological examination of biopsies positive for the CD1a antigen and treated at the Division of Hematology–Oncology, Department of Pediatrics, Phramongkutklao Hospital, a 1200 bed military teaching hospital for the Phramongkutklao College of Medicine, and tertiary referral center for childhood cancer. Data from patients without a confirmed diagnosis by histopathology, who had not undergone imaging, or were not treated primarily at Phramongkutklao Hospital, were excluded.

For all patients, demographic data, imaging studies, type of treatment, and treatment outcomes were also reviewed.

### Operational definition

Patients were classified according to the Histiocyte Society LCH-IV guidelines [24], which consider the number of system involvements, number of lesions, site of involvement, and whether the disease involves a “risk-organ” (the hematopoietic system, liver, or spleen) [10]. The 5 classifications are bullet pointed below.

SS: one system involvement and RO−

- SS, unifocal: one system involvement with one lesion
- SS, multifocal: one system involvement with at least two lesions
- MFB, bone involvement only with at least two lesions

MS: more than two systems involved

- MS, RO− or low risk: more than two systems RO−
- MS and RO+ or high risk: more than two systems with at least one organ of risk involvement or any organ of risk involvement, regardless of the number of organs involved

Remission of disease was defined as resolution of all signs and symptoms. Progressive disease was defined as progression of signs or symptoms and/or appearance of new lesions. Reactivation was defined as the reappearance of signs and symptoms of active disease after either complete disease resolution or a period of disease control that persisted for >3 months on maintenance therapy.

### CMT protocols

Patients were treated as per institutional protocols based on the oncologists’ expertise. CMT regimens were mainly based on International Collaborative Treatment Protocol for Children and Adolescents with LCH included LCH-III study (vinblastine [VBL], prednisolone (Pred), 6-mercaptopurine [6-MP], and methotrexate [MTX]) [18], LCH-IV study (frontline with VBL, Pred and 6-MP, and salvage regimens) [24], DAL-Hx83 protocol (VBL, etoposide, 6-MP, MTX, and Pred) [25, 26], and individualized treatment protocols such as cyclophosphamide (CTX), vincristine (VCR), cytosine arabinoside (Ara-C), and Pred.

### Statistical analysis

Clinical characteristics, imaging studies, and treatments were calculated as descriptive statistics with mean and standard deviation or as median (range) for continuous variables, and frequency and percentage for categorical variables. Survival was calculated using a Kaplan–Meier method and comparing using a Cox proportional hazard model. Statistical analysis was performed using STATA/MP (version 12; STATA Corp.) with *P* < 0.05 considered significant.

## Results

### Patient characteristics

Patient characteristics included age, sex, DI, and organ system involvement; these were analyzed for 14 patients who all met eligible criteria and whose data were included in the study (**Table 1**). The patient's ages ranged from 0.25 to 15.6 with a median age of 2.5 years. Girls were more predominant than boys at a ratio of 3.7:1, and 14% (2/14) of patients had DI at diagnosis. All patients with LCH who had DI at diagnosis had pituitary involvement. Six (43%) patients had SS involvement, which mostly (3 patients, 50%) presented with single bone involvement. Five patients (62%) with MS involvement had RO+ at diagnosis. Among patients with RO−, the involved systems included the pituitary, bone, skin, and lymph nodes (**Table 2**). The median follow-up time was 6.8 years.

**Table 1:** Patient demographic and clinical characteristics

**Demographic**	**n = 14 (%)**
Age at diagnosis (years)	
Median (range)	2.5 (0.25–15.6)
Mean ± SD	5.1 ± 4.8
Sex	
Female	11 (79)
Male	3 (21)
DI at diagnosis	2 (14)
System involvement	
SS	6 (43)
Skin only	1 (17)
Single bone	3 (50)
MFB	2 (33)
MS	8 (57)
Low risk	3 (38)
High risk	5 (63)
Follow-up (years)	
Median (range)	6.8 (0.5–16.2)
Mean ± SD	6.9 ± 5.7

DI, diabetes insipidus; MFB, multifocal bone; MS, multisystem; SS, single system.

**Table 2:** Characteristics and outcomes of patients with LCH (n = 14)

**No**	**Age at diagnosis (years)**	**Sex**	**System involvement (SS vs. MS)**	**Organ/system involvement**	**Organ/system of risk involvement**	**DI at diagnosis**	**Treatment**	**CMT protocols**	**Disease status**	**Status at last follow-up**	**Follow-up (years)**
**1**	1.7	Female	MS	Skin, bone	Liver, spleen, hematopoietic	No	CMT	Ara-C, Pred	Progressive	DWD	1
**2**	0.25	Female	MS	Lung, bone	Liver, spleen, hematopoietic	No	CMT	DAL-Hx83	Remission	ANED	16
**3**	1.2	Female	MS	Bone, LN	Liver, spleen	No	CMT	LCH-III	Reactivation MFB	AWD	8.6
**4**	15.6	Male	MS	Lung, MFB (vertebrae)	Liver, spleen	No	CMT	LCH-IV	Reactivation	AWD	2.7
**5**	1.3	Female	MS	Skin, bone	Liver, spleen, hematopoietic	No	CMT	LCH-IV	Progressive	DWD	0.6
**6**	9	Female	SS	Bone	–	No	RT, CMT	CTX, VCR, Pred	Remission	ANED	12
**7**	0.5	Female	SS	Skin	–	No	Observation	–	Remission	ANED	12
**8**	3	Female	SS	Bone (CNS risk)	–	No	Curettage, CMT	LCH-III	Remission	ANED	15
**9**	8	Female	MS	MFB, pituitary	–	Yes	CMT	LCH-III	Remission	ANED	3
**10**	7	Female	SS	Bone	–	No	Surgery	–	Remission	ANED	1
**11**	10	Male	SS	MFB	–	No	CMT	LCH-III	Remission	ANED	6
**12**	2	Female	MS	Skin, pituitary, bone	–	Yes	CMT	LCH-III	Remission	ANED	9
**13**	10.25	Male	MS	Bone (vertebrae), LN	–	No	Surgery, CMT	LCH-III	Remission	ANED	8
**14**	1.4	Female	SS	MFB	–	No	CMT	LCH-IV	Remission	ANED	0.5

ANED, alive with no evidence of disease; Ara-C, cytosine arabinoside; AWD, alive with disease; CMT, chemotherapy; CNS, central nervous system; CTX, cyclophosphamide; DI, diabetes insipidus; DWD, died with disease; LCH, Langerhans cell histiocytosis; LN, lymph nodes; MFB, multifocal bone; MS, multisystem; Pred, prednisolone; RT, radiation; SS, single-system; VBL, vinblastine; VCR, vincristine.

### MS and RO+ patients

Characteristics of patients with RO+ are shown in **Table 3**. All of the RO+ patients had at least 2 RO+ (liver and spleen). Three of 5 (60%) of RO+ patients had 3 RO+ (the liver, spleen, or hematopoietic system) with concomitant abnormal liver function test (LFT) in terms of hypoalbuminemia or hyperbilirubinemia. All patients had been treated with an intensified CMT regimen for LCH including VBL, Pred, and 6-MP except for patient No. 1 who had hyperbilirubinemia, which was a contraindication for VBL. Only 1 patient (No. 2) achieved complete remission of the disease without disease reactivation and was still alive without evidence of disease after follow-up for 16 years. Two patients (Nos. 1 and 5) experienced disease progression and died with the disease within 1 year after diagnosis. Both patients had disease reactivation in the hematopoietic system and liver. Of the 2 patients who were RO+, without hematopoietic involvement, 1 had MFB relapse after end of therapy for 18 months (30 months after diagnosis) (No. 3) and 1 had disease reactivation during the treatment (after 12 months from diagnosis) (No. 4). Both patients are still alive with disease (AWD) and on treatment. Patient No. 3 has the disease controlled with MTX and 6-MP. Patient No. 4 who had liver, lung, and bone disease reactivation, has the disease controlled by Pred, Ara-C, and CTX.

**Table 3:** MS “risk” LCH (RO+ MS-LCH) characteristics of 5 patients

**No**	**Age at diagnosis (years)**	**Sex**	**Abnormal LFT**	**Organ/system of risk involvement**	**Other organ involvement**	**CMT regimen**	**Disease status**	**Status last follow-up**	**Follow-up (years)**	***BRAF*^V600E^ mutation**
**1**	1.7	Female	TP 4.8 Alb 2.4 TB 13.7 DB 12.9 AST 56 ALT 47 ALP 451	Liver, spleen, hematopoietic	Skin, bone	Ara-C, Pred	Progressive	DWD	1	Not done
**2**	0.25	Female	TP 8 Alb 4.8 TB 14 DB 0.5 AST 30 ALT 16 ALP 254	Liver, spleen, hematopoietic	Lung, bone	DAL-Hx83: VBL, VP-16, 6-MP, MTX, Pred	Remission	ANED	16	Not done
**3**	1.2	Female	–	Liver, spleen	Bone, LN	LCH-III (High risk): MTX, VBL, Pred, 6-MP	Reactivation MFB	AWD	8.6	Not done
**4**	15.6	Male	–	Liver, spleen	Lung, bone (special site)	LCH-IV adapted: (High risk): VBL, Pred, 6-MP	Reactivation	AWD	2.7	Negative
**5**	1.3	Female	TP 6.27 Alb 2.73 TB 0.65 DB 0.28 AST 29.8 ALT 19.2 ALP 80	Liver, spleen, hematopoietic	Skin, bone	LCH-IV adapted: (High risk): VBL, Pred, 6-MP	Progressive	DWD	0.6	Positive

6-MP, 6-mercaptopurine; ANED, alive with no evidence of disease; Alb, albumin; ALP, alkaline phosphatase; ALT, alanine aminotransferase; Ara-C, cytosine arabinoside; AST, aspartate aminotransferase; AWD, alive with disease; CMT, chemotherapy; DB, direct bilirubin; DWD, died with disease; LCH, Langerhans cell histiocytosis; LFT, liver function test; LN, lymph nodes; MFB, multifocal bone; MS, multisystem; MTX, methotrexate; Pred, prednisolone; RO+, risk-organ involvement; TB, total bilirubin; TP, total protein; VBL, vinblastine; VP-16, etoposide.

### Radiological studies

Eleven of 14 patients (79%) showed an osteolytic lesion of the skull or widening of the sella turcica as observed from the skull radiograph (**Table 4**). Eight of 11 patients (73%) were further evaluated with brain MRI. Only 1 patient (13%) demonstrated normal MRI findings. Involvement of the pituitary gland and stalk was identified in 2 patients. The remaining patients showed masses with bony destruction.

**Table 4:** Radiology of patients with LCH (n = 14)

**Imaging**	**n = 14 (%)**
**Skull X-ray**	
Normal	3 (21)
Abnormal	11 (79)
**MR brain**	8 (73)
Normal	1 (13)
Abnormal	7 (88)
**MR brain not done**	3 (27)
**Tc-99m bone**	
Done	9 (64)
Not done	5 (36)

**FDG-PET-CT**	
Done	4 (29)
Not done	10 (71)

CT, computed tomography; FDG, fluorodeoxyglucose; LCH, Langer-hans cell histiocytosis; MR, magnetic resonance; PET, positron emission tomography.

Subsequently, a Tc-99m bone imaging was conducted for 9 patients (64%), demonstrating abnormal uptake in 8 (89%). One patient showed normal uptake on Tc-99m bone imaging with multiple osteolytic lesions from the whole-body skeletal survey.

FDG-PET-CT was performed for 4 patients (29%) and demonstrated FDG-avid lesions. Three of 4 patients received both Tc-99m bone and FDG-PET-CT imaging, which had parallel positive results. Only 1 patient underwent FDG-PET-CT imaging and a whole-body skeletal survey without Tc-99m bone imaging, and both types of imaging showed abnormal results.

### Type of treatment

Surgery was performed for 3 (21%) patients (**Table 5**). One patient with a single bone lesion presented with otitis media with a diagnosis in 1998, which had been curettaged, and was subsequently treated with LCH-III adjuvant CMT protocol. Another patient with single bone involvement had the bone partially resected. One with RO− MS-LCH presenting spinal cord compression underwent a partial laminectomy with LCH-III adjuvant CMT protocol.

**Table 5:** Treatment and outcomes of patients with LCH (n = 14)

**Treatment**	**n = 14 (%)**
**Surgery**	3 (21)
Curettage	1 (33)
Partial resection	2 (67)

**CMT**	12 (86)
**SS**	4 (33)
Single bone	2 (50)
MFB	2 (50)
**MS**	8 (67)
Low risk	3 (38)
High risk	5 (62)

**RT**	1 (7)

**Disease status**	
Remission	10 (71)
Reactivation/progressive	4 (29)
On therapy	3 (75)
Off therapy	1 (25)

**Status at last follow-up**	
ANED	10 (72)
AWD	2 (14)
DWD	2 (14)

ANED, alive with no evidence of disease; AWD, alive with disease; CMT, chemotherapy; DWD, died with disease; LCH, Langerhans cell histiocytosis; MFB, multifocal bone; MS, multisystem; RT, radiation therapy.

Twelve patients (86%) received CMT. One patient with single bone involvement presented a gingival mass with a diagnosis in 1997, and received CMT consisting of CTX, VCR, and Pred with concurrent radiotherapy. Nine patients (75%) received VBL-containing CMT (**Table 3**).

### Outcomes of treatment

Ten of 14 (71%) patients experienced disease remission and remained alive with no evidence of disease (ANED). Four (29%) patients with RO+ complicated with progressive or reactivation of disease. Two (14%) patients with disease reactivation survived with the disease after treatment. However, 2 (14%) patients had refractory disease and eventually died with the disease (**Table 5**).

**Figure 1:**
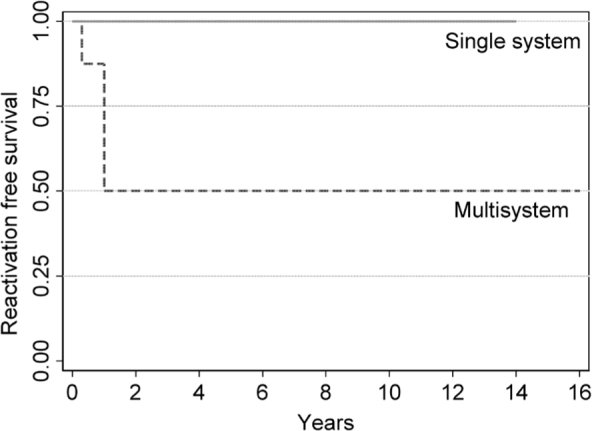
Five-year reactivation EFS for patients with LCH and SS (n = 6, solid line) and MS (n = 8, dashed line) involvement (*P* = 0.085). Survival was calculated using a Cox proportional hazard model. EFS, event-free survival; LCH, Langerhans cell histiocytosis; MS, multisystem; SS, single system.

**Figure 2:**
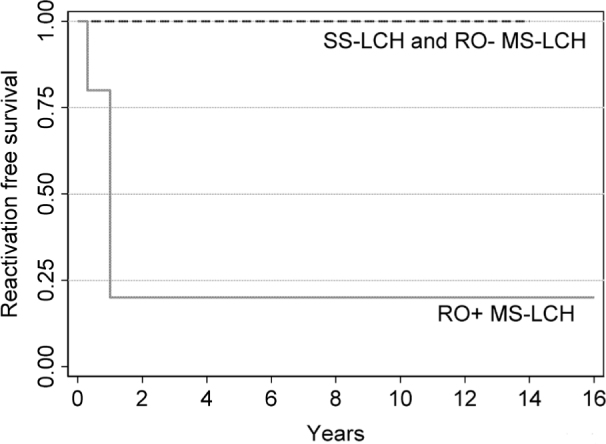
Five-year reactivation EFS for patients with LCH and RO+ (n = 5, solid line) and RO− (n = 9, dashed line) (*P* = 0.005). Survival was calculated using a Cox proportional hazard model. EFS, event-free survival; LCH, Langerhans cell histiocytosis; MS, multisystem; RO−, no risk-organ involvement; RO+, risk-organ involvement; SS, single system.

Five-year event-free survival (EFS) for patients with SS-LCH tended to be better than MS-LCH (100% vs. 50%, *P* = 0.085, **Figure 1**). However, 5-year EFS for patients with RO+ MS-LCH was worse than those having RO− MS-LCH and SS-LCH (20% vs. 100%, *P* = 0.005, **Figure 2**).

## Discussion

In the present study, we describe the clinical characteristics, imaging modalities, treatment regimens, and outcomes for children with LCH in a large tertiary care center for pediatric oncology in Bangkok, Thailand over a 20-year period. The incidence of LCH is approximately <6 per million persons per year in Thailand [8]. This is consistent with other countries in Asia with 3 patients per year [27] and 5–9 per million persons per year in Europe [28]. During the 20-year period of the study to the end of 2016, we found only 14 LCH patients, due to the rarity of disease. Only 14% of patients had DI, consistent with a related report of from 2% to 25% of patients with LCH in developed countries [12, 29]. However, 2 patients with LCH presenting with DI had been classified as RO− MS-LCH and 1 of these experienced MFB involvement. A related report by Imashuku et al. [19] showed MS-LCH with MFB was associated with a significantly higher incidence of DI.

The most common organ involvements for LCH are bone [12] and skin [30]. Therefore, plain X-ray radiographs [31, 32], skeletal surveys, and Tc-99m bone imaging [33] are crucial modalities and investigations of choice for patients with LCH to explore systematic involvement of the disease. We found that almost 80% of patients with LCH had been diagnosed after plain X-ray radiographs of the skull. In addition, we found Tc-99m bone imaging showed abnormal results in almost 90% of patients, and 100% for FDG-PET-CT. Tc-99m bone imaging is currently substituted by FDG-PET-CT scan [34] because it is more highly sensitive for detecting positive lesions with a very low false-positive rate among patients with LCH [14].

**Figure 3:**
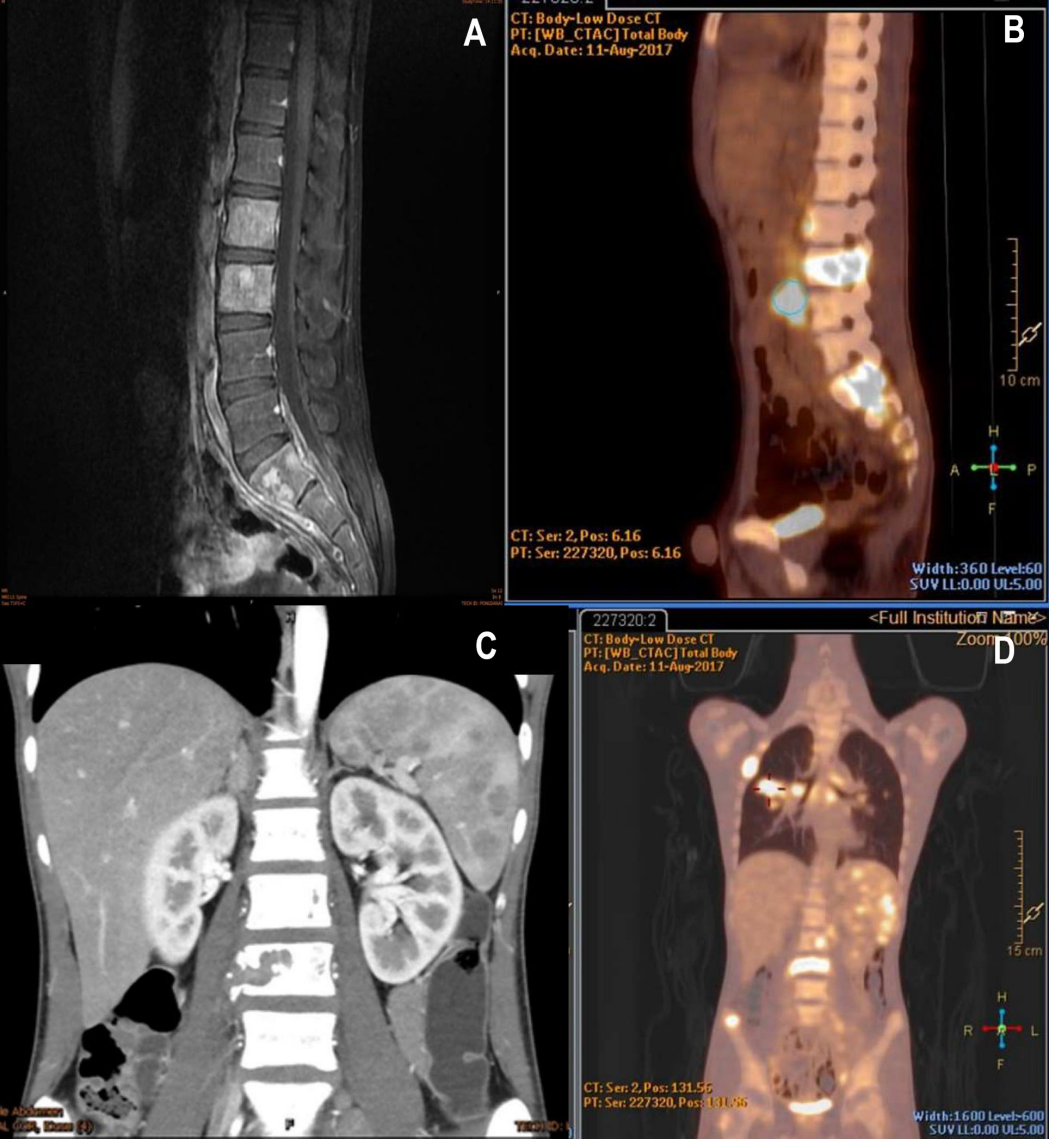
Comparison of CT/MRI and FDG-PET-CT scan from patient No. 4 in **Table 2**. **(A)** Sagittal T1 postcontrast MRI demonstrating abnormal marrow signal intensity involving L2, L3, S1–S2 vertebral bodies **(B)** paralleled with the FDG-PET-CT image. **(C)** Contrast enhanced CT image of the abdomen demonstrating hepatosplenomegaly with heterogenous contrast enhancement and scattered hypodense lesions in the spleen and a lytic lesion at the L3 vertebral body **(D)** corresponding FDG-PET-CT image confirming hypermetabolic activities at L2–L3 vertebral bodies and the spleen. Additional hypermetabolic activities are noted at the right sacral alar, right hilar lymph node, right middle lobe, and right-sided of second rib, which is suggestive of disease involvement. CT, computed tomography; FDG, fluorodeoxyglucose F18; MRI, magnetic resonance imaging; PET, positron emission tomography.

Interestingly, patient No. 4 with RO+ MS-LCH was 15 years old at diagnosis. He had liver, spleen, lung, vertebral, and multiple bone involvement indicated by CT and MRI. As the age is uncommon for LCH and CT/MRI was unable to distinguish active LCH lesions and other causes, FDG-PET-CT was also performed. The result showed hypermetabolic activities in active lesions, which were not clearly indicated by MRI or CT (**Figure 3**). In our present study, 3 patients received both Tc-99m bone imaging and FDG-PET-CT determining parallel positive results in both studies. This implied that the sensitivity of the FDG-PET-CT is very high for detecting LCH lesions compared with Tc-99m bone imaging and plain X-ray radiographs as described by Phillips et al. [34]. Moreover, FDG-PET-CT can be more useful to monitor and evaluate activity of the disease after treatment than Tc-99m bone imaging because the radiotracer uptake in Tc-99m bone imaging might persist over months to years even without active disease. However, only a small number of patients underwent FDG-PET-CT. Further exploration of the effectiveness of this imaging is warranted.

Treatment of LCH is tailored by organ system involvement. SS-LCH would be cured by local treatment such as curettage or surgery [35]. Low-intensity CMT in MFB SS-LCH [36] and RO− MS-LCH [18] have shown excellent outcomes. By contrast, disease remission and prognosis of RO+ MS-LCH is still unsatisfactory even using high-intensity therapy and prolonged therapy regimens [18].

The outcome of pediatric LCH in Thailand is consistent with that in developed countries in the Asia–Pacific such as Taiwan. Five-year EFS was 100% in SS-LCH for both countries. However, 5-year EFS of MS-LCH was lower in Thailand (50%) than it was in Taiwan (85.7%) [27]. Outcomes for RO+ MS-LCH are inferior to RO− MS-LCH and SS-LCH [18] especially correlated with the number of RO+. Hematopoietic involvement was associated with a worse prognosis in RO+ [37]. From our present study, patients with MS-LCH having 2 RO+; liver and spleen involvement (patient Nos. 3 and 4; **Table 2**) were still alive with evidence of disease reactivation, while patients with 3 RO+ (Nos. 1 and 5) died from disease progression. Patient No. 2 with 3 RO+ was ANED. This patient had not undergone a bone marrow examination, but still met the criteria of hematopoietic involvement due to anemia and thrombocytopenia. Bicytopenia might be due to other causes, i.e., an infectious process, which might not be truly explained by bone marrow involvement in LCH. We found patients with RO+ MS-LCH (Nos. 1 and 5), who had hypoalbuminemia and/or conjugated hyperbilirubinemia, but had disease progression and subsequently died from the disease. Conjugated hyperbilirubinemia could be a sign of sclerosing cholangitis with greater mortality for MS-LCH [12, 38]. Braier et al. [37] reported that hypoalbuminemia was associated with inferior prognosis in MS-LCH. By contrast, patients having disease remission, with LFT, had only abnormal unconjugated hyperbilirubinemia.

Historical treatments such as the DAL-Hx83 protocol, conducted between 1983 and 1991 in Europe, were an effective regimen especially for RO+ MS-LCH due to its high intensity [25, 26]. Currently, standard treatment of RO+ MS-LCH for Thai children has been adapted from the Histiocyte Society LCH studies. However, the survival rate for RO+ MS-LCH in our present study was approximately 20% by comparison with 84% in the LCH-III study [12, 18]. This might be the result of the small number of 5 patients in our study compared with the 285 patients in the LCH-III study as well as limited resources for complex supportive care in patients receiving high-intensity CMTs with vital organ damage in RO+ MS-LCH.

Disease reactivation is common in patients with LCH patients. Patients with MFB disease reactivation usually respond well to second-line therapy including oral 6-MP and MTX [15] as did patient No. 3 (**Table 3**). By contrast, prognosis is dismal in patients with disease progression in risk organs during therapy especially in those with hematopoietic and liver involvement. More intensified CMT with high-dose Ara-C may be effective for achieving disease remission [39] as it was in patient No. 4 (**Table 3**). Moreover, BRAF inhibitors are promising therapeutic regimen for this group of patients [40].

*BRAF*^V600E^ activating mutation has been identified in 70% of patients with LCH of myeloid origin [12]. Therefore, *BRAF*^V600E^ activating mutation is an option to confirm diagnosis of LCH in equivocal settings. Because a BRAF inhibitor demonstrated efficacy in LCH [41], detected *BRAF*^V600E^ activating mutation in LCH may facilitate the feasibility of using a BRAF inhibitor as a second-line treatment. In our setting, *BRAF*^V600E^ activating mutation was analyzed in 2 patients with RO+ MS-LCH and detected in 1 (patient No. 5) who had progressive disease despite using intensive CMT. This finding corresponded with those of Bhatia et al. who reported that *BRAF*^V600E^ activating mutation in childhood LCH correlated with MS-LCH and poor survival [5]. A multicenter prospective study combining genetic testing of *BRAF*^V600E^ activating mutation could be modified with clinical correlation particularly in an MS-LCH population. BRAF inhibitors would provide a new generation treatment to improve outcomes in this group of patients.

To our knowledge, this study is the first to record the clinical characteristics and outcomes of LCH in Thailand over 20 years. Considering the rarity of disease, variety of clinical presentations, and serious comorbidities in RO+ MS-LCH, it is our opinion that a uniformed CMT protocol and centralized care by disease-specific professionals would facilitate an optimal standard of care that would result in better outcomes.

## Limitations

This was a retrospective study in which some data was unavailable and might not represent the entire patient population of our institute. Moreover, the study duration was short for some patients. Patients who had not been followed at the Division of Hematology–Oncology, such as those with skin-only LCH, who were treated at the Dermatology Department, were not included in the study. These might have an effect on outcomes for the small sample size. A multicenter prospective study would improve the power and quality of data, which would more truly represent the LCH disease situation in Thailand.

## Conclusions

LCH is a rare disease with some patients having DI. FDG-PET-CT is considered as an investigation of choice and has greater accuracy than conventional plain radiographs and Tc-99m bone imaging to detect sites of active disease and for disease monitoring after treatment. SS-LCH and RO− MS-LCH have been successfully treated using local treatment or low-intensity CMT. However, patients with RO+ MS-LCH have often encountered disease reactivation or progressive disease with a dismal prognosis. Hematopoietic system and organ of risk involvement were associated with worse prognosis. Hypoalbuminemia and conjugated hyperbilirubinemia may predict a greater mortality in RO+ MS-LCH.
